# Supplementary material for: Complexome profiling of the Chlamydomonas psb28 mutant reveals TEF5 as an early PSII assembly factor
Source: Plant Cell. 2025 Mar 17;37(6):koaf055. doi: 10.1093/plcell/koaf055 (PMC12164590; doi:10.1093/plcell/koaf055)
Supplement: koaf055_Supplementary_Data [file koaf055_supplementary_data.zip › psb28_interactive.zip!/plots/intensity/NFS1_Cre02.g146250.t1.2.html]

|  |  |
| --- | --- |
| **Identifier** | Cre02.g146250 |
| **Trivial name** | NFS1 |
| **Euclidean distance** | 13773.95 |
| **Average intensity sum (WT)** | 63.1 || **Average intensity sum (Mut)** | 523.0 |
| **Adjusted euclidean distance** | 26.33 |
| **MapMan annotation** | amino acid metabolism.synthesis.serine-glycine-cysteine group.cysteine |
| **p-value** (Welch test) of intensity sums | 0.32778 |
